# Supplementary material for: From data to diagnosis: An innovative approach to epilepsy prediction with CGTNet incorporating spatio-temporal features
Source: PLoS One. 2025 Dec 2;20(12):e0337007. doi: 10.1371/journal.pone.0337007 (PMC12671784; doi:10.1371/journal.pone.0337007)
Supplement: Attachment — Submitted filename: PONE-D-24-43275_reviewer-comments.docx [file pone.0337007.s001.docx]

**Review of Paper PONE-D-24-43275**

**Title:**

**From data to diagnosis: an innovative approach to epilepsy prediction with CGTNet incorporating spatio-temporal features**

**Summary:**

This paper presents CGTNet, a novel deep learning architecture designed to predict epileptic seizures using EEG data. CGTNet combines multi-scale convolutional networks, gated recurrent units (GRUs), and Sparse Transformers to effectively capture spatiotemporal features from EEG signals. Evaluations on the CHB-MIT and SWEC-ETHZ datasets demonstrate its high performance, with 98.89% accuracy, 98.52% sensitivity, 98.53% specificity, an AUROC of 0.97, and an MCC of 0.975. These results underline the model's technical innovations and the potential of AI for epilepsy monitoring and early detection, contributing to advancements in healthcare technology.

**Strengths And Weaknesses:**

## Strengths

- A very complete introduction.
- It has a thorough explanation of the main idea.
- Clearly explain the methodology.
- Results are perfectly described and used proper evaluation metrices.

## Weaknesses

Overall, the writing can be significantly improved to address the following concerns.

- **Dataset:**
  1. For CHB-MIT dataset you mentioned that it is totaling approximately 916 hours of EEG recording, but in table 2 the sum of them is 518.6 hours.
  2. In table 2, if the second column is the number of channels why it is not integer in some case (like patient 20 is 23.3 and patient 24 is 12.3)?
  3. In line 211, you mentioned that patients 12 and 20 from CHB-MIT were excluded. You would explain the reason clearly (for example: high noise).
  4. Did you include patient 24 of CHB-MIT data set? If yes, please fix line 213.
- **Method:**
  1. You would add the down sampling part of preprocessing in your algorithm (table 4), since your EEG has different sampling rates.

**Questions:**

Answering the following questions and addressing the weaknesses above can significantly improve my score.

1. Since you used EEG data with different sampling rate, what is your final sampling rate to prepare EEG as input for the model? (line 301)
