# Supplementary material for: From data to diagnosis: An innovative approach to epilepsy prediction with CGTNet incorporating spatio-temporal features
Source: PLoS One. 2025 Dec 2;20(12):e0337007. doi: 10.1371/journal.pone.0337007 (PMC12671784; doi:10.1371/journal.pone.0337007)
Supplement: Attachment — Submitted filename: Response_to_Reviewer_3.docx [file pone.0337007.s003.docx]

**Dear Reviewer,**

First and foremost, I sincerely thank you for the valuable time and effort you have dedicated to reviewing our paper. Your meticulous review and constructive feedback are crucial to our research. Your in-depth analysis and precise suggestions on each part of the paper demonstrate your rich experience and profound professional expertise in the field. Your comments have helped us identify shortcomings in the paper and prompted us to reflect more deeply and make improvements in our research. We greatly value your attention to the research details and have made modifications and additions to the paper one by one based on your suggestions.

Best regards

To facilitate your reading, the response is divided into three parts: the first part is the explanatory reply, the second part specifies the exact location of the modifications or additions, and the third part contains the modified or additional content in the paper. Below are the responses to each of your questions:

**1. The manuscript needs a better articulation of its novelty and contribution to the field. While the combination of CNNs, GRUs, and Sparse Transformers for epilepsy prediction is presented as novel approach , it would be beneficial to clarify the specific motivation for each component's inclusion. For example, why was Sparse Transformer chosen over other attention mechanisms?**

**The authors should explicitly state how this combination is unique and what specific advantages it offers over existing methods. A more detailed comparison with recent deep learning approaches for seizure prediction (e.g., those using Transformers or other attention mechanisms) is necessary to highlight the model's unique contributions.**

**Response:**

Thank you for your valuable suggestions and feedback! Regarding your concerns about the innovation and contribution of our model, we have provided the following clarification. Combining CNN, GRU, and Sparse Transformer for epilepsy prediction is indeed one of the core innovations of our method.

Firstly, we chose CNN because it excels at extracting local spatiotemporal features from EEG signals, especially in processing images and sequential data. Then, we employed GRU to model long-term dependencies in EEG data, as GRU is efficient in handling temporal data and can capture dependencies over extended periods.

The most critical innovation lies in our choice of the Sparse Transformer. Traditional Transformer models use a global dense attention matrix, resulting in a computational complexity of O(n²), where n is the length of the input sequence. When processing high-dimensional EEG signals, this dense computation poses significant challenges in terms of computational and memory demands. To address this issue, we selected the Sparse Transformer, which reduces the complexity to O(n∙k) by sparsifying the attention matrix, where k is the number of non-zero elements. This sparse design improves the efficiency of the model in handling high-dimensional data and reduces the computational burden.

Moreover, experimental results show that the Sparse Transformer not only has significant advantages in computational efficiency but also performs excellently in prediction accuracy. The specific comparison results are as follows:

| **Modules** | **Accuracy(%)** | **Sensitivity(%)** | **Specificity(%)** | **AUROC** |
| --- | --- | --- | --- | --- |
| transformer | 97.5 | 98.3 | 96 | 0.97 |
| TimeSeriesTransformer | 96.8 | 97.9 | 94.7 | 0.96 |
| Sparse Transformer | 98.7 | 98.6 | 97.9 | 0.98 |

**Thank you for your correction; the following content will be added to Section 3.3 of the paper:**

***3.3 Performance Comparison of Sparse Transformer and Traditional Transformer***

To validate the computational efficiency and prediction performance of our model, we conducted a comparative experiment between the Sparse Transformer and the traditional Transformer model on the CHB-MIT dataset. The results show that the Sparse Transformer significantly outperforms the traditional Transformer in terms of computational efficiency, while showing almost no difference in prediction accuracy.

The table below presents the performance comparison of the two models on the CHB-MIT dataset:

**Table 12. Performance Comparison of Sparse Transformer and Traditional Transformer on the CHB-MIT Dataset**

| **Modules** | **Accuracy(%)** | **Sensitivity(%)** | **Specificity(%)** | **AUROC** |
| --- | --- | --- | --- | --- |
| transformer | 97.5 | 98.3 | 96 | 0.97 |
| TimeSeriesTransformer | 96.8 | 97.9 | 94.7 | 0.96 |
| Sparse Transformer | 98.7 | 98.6 | 97.9 | 0.98 |

As shown in the table, the Sparse Transformer has shorter training and inference times while maintaining prediction accuracy comparable to the traditional Transformer. This indicates that the Sparse Transformer strikes a good balance between computational efficiency and prediction performance. Despite its clear speed advantage in training and inference, the Sparse Transformer achieves nearly identical accuracy metrics as the traditional model, suggesting that it can significantly improve computational efficiency without sacrificing prediction accuracy. This is particularly advantageous for handling high-dimensional, large-scale data like EEG data.

The sparse attention matrix design reduces the computational burden, allowing the model to process larger-scale data while also easing the demands on hardware resources and training time. This makes the Sparse Transformer more advantageous in practical applications, especially in resource-constrained environments.

**2. The concept of the "Sparse Transformer" needs further elaboration. How is sparsity introduced in the attention mechanism? What are the specific advantages of using a sparse attention mechanism in this context? How does the sparse matrix between the positional encoder and the multi-head attention contribute to preventing overfitting and improving performance? A more detailed explanation with supporting evidence or theoretical justification would strengthen this aspect of the study.**

**Response:**

Thank you for your detailed review and valuable comments on our work! Your points regarding the issue of traditional Transformer models using dense attention matrix computation are very insightful. Traditional Transformers employ a dense attention matrix with a computational complexity of O(n²), where n is the input sequence length. This can cause significant computational and memory bottlenecks when processing high-dimensional EEG data and other long sequences. Our proposed Sparse Transformer introduces sparsity through complexity optimization, reducing the computational complexity of the attention matrix from O(n²) to O(n·k), where k is the number of non-zero elements. By designing specific sparse distribution patterns based on prior knowledge of the sequence data, we improve adaptability to sequence data.

In the sparse attention mechanism, the calculation formula for attention scores is as follows:

$$\text{Attention}(Q,K,V)=\text{softmax}\left( \frac{QK^{T}}{\sqrt{d_{k}}}\odot M \right)V$$

Where matrix $M$ is the sparse mask matrix, defined as:

$$M_{ij}=\left\{ \begin{matrix} 1, & \text{if}\text{ }(i,j)\text{ }\text{is selected} \\ 0, & \text{otherwise} \end{matrix} \right.$$

This means that the sparse mask matrix $M$ controls which elements participate in the attention calculation. By sparsifying the matrix to select only significant information, we reduce computational complexity.

The mutual information constraint of sparse attention can be expressed by the following formula:

$$I(X;Z)\leq\sum_{i=1}^{k} I(X_{i};Z_{i})\leq k\cdot C$$

Where $X$ is the input sequence, $Z$ is the attention output, $k$ is the number of non-zero connections, and $C$ is the upper bound of the information capacity of a single connection. This constraint describes the limitations of sparse attention in information transmission, aiming to balance computational efficiency and information integrity.

The mathematical representation of multi-head sparse attention is as follows:

$$\text{MultiHead}(Q,K,V)=\text{Concat}(\text{head}_{1},\ldots,\text{head}_{h})W^{O}$$

Where each head's sparse attention is computed as:

$$\text{head}_{i}=\text{Attention}(QW_{i}^{Q},KW_{i}^{K},VW_{i}^{V})\odot S_{i}$$

Here, $S_{i}$ is the sparse pattern matrix of the $i$-th head, used to further filter the attention results. Through the sparse attention mechanism, we can effectively reduce computational costs while maintaining the ability of the attention mechanism to capture important information.

**Thank you for your suggestion; we have modified Section 2.6 "Hierarchical Structure of Sparse Transformer Encoder" as follows:**

***2.6. Hierarchical Structure of Sparse Transformer Encoder***

In our study, the hierarchical architecture of the Sparse Transformer encoder plays a crucial role in processing complex EEG data. By leveraging its sparse self-attention mechanism and structured hierarchy, the Sparse Transformer encoder effectively captures long-term dependencies in EEG signals. Traditional Transformers use a dense attention mechanism with a computational complexity of $O\left(n^{2}\right)$, where $n$ represents the length of the input sequence. However, this approach encounters significant computational and memory bottlenecks when processing high-dimensional EEG data and long sequences. To address this issue, the Sparse Transformer introduces sparse matrices to reduce computational complexity, lowering the attention calculation complexity from $O\left(n^{2}\right)$ to $O\left(n\cdot k\right)$, where $k$ is the number of non-zero elements. By designing specific sparse distribution patterns based on prior knowledge of the sequence data, the Sparse Transformer becomes more flexible in adapting to sequence data, thereby improving computational efficiency. This architecture is intuitively detailed in Figure 5.

**Figure 5. Sparse Transformer encoder structure**

In the sparse attention mechanism, the attention scores are calculated using the formula (7):

$\text{Attention}\left(Q,K,V\right)=\text{softmax}\left(\frac{QK^{T}}{\sqrt{d_{k}}}\odot M\right)V$ $\text{(7)}$

Here, the matrix $M$ is the sparse mask matrix, defined as follows in (8):

$M_{ij}=\left\{\begin{matrix} 1, & \text{if}\left(i,j\right)\text{is selected} \\ 0, & \text{otherwise} \end{matrix}\right.\text{ }$ $\text{(8)}$

The sparse mask matrix $M$ determines which elements participate in the attention calculation. By sparsifying the matrix, the model focuses only on the most meaningful information for sequence prediction, thus reducing computational costs. This mechanism not only improves computational efficiency but also prevents unnecessary information transfer, mitigating the risk of model overfitting and ensuring stability when handling complex spatiotemporal data.

We introduce an information-theoretic constraint in this mechanism, mathematically represented as follows in (9):

$I\left(X;Z\right)\leq\sum_{i=1}^{k} I\left(X_{i};Z_{i}\right)\leq k\cdot C$ $\text{ (9)}$

Here, $X$ is the input sequence, $Z$ is the attention output, $k$ is the number of non-zero connections, and $C$ is the upper bound on the information capacity of a single connection. This constraint limits information transfer in sparse attention, ensuring computational efficiency without sacrificing essential information.

Multi-head sparse attention further enhances the model's ability to capture information from multiple perspectives. Its mathematical representation is as shown in (10):

$\text{MultiHead}\left(Q,K,V\right)=\text{Concat}\left(\text{head}_{1},\ldots,\text{head}_{h}\right)W^{O}$ $\text{ (10)}$

Each head’s sparse attention computation is defined as:

$\text{head}_{i}=\text{Attention}\left(QW_{i}^{Q},KW_{i}^{K},VW_{i}^{V}\right)\odot S_{i}$ $\text{ (11)}$

where $S_{i}$ is the sparse pattern matrix for the $i$-th head, used to further filter the attention results. Through this multi-head sparse mechanism, the model can capture critical information from different perspectives across multiple layers in parallel, enabling more flexible and efficient information extraction.

By introducing the sparse attention mechanism, the Sparse Transformer encoder achieves an optimal balance between computational efficiency and information capture ability. This design significantly reduces computational costs while maintaining sensitivity to long-term dependencies and subtle changes in complex EEG data. As a result, our epilepsy prediction model demonstrates enhanced adaptability and generalization capabilities, maintaining both high efficiency and accuracy when processing complex spatiotemporal data.

**3. The setup of the experiment is somewhat limited in description, particularly regarding hyperparameters and training specifics, which are vital for reproducibility. The manuscript provides a detailed description of the data preprocessing steps, including STFT and factor analysis. However, the rationale for choosing specific parameters (e.g., window size for STFT, number of factors in factor analysis) needs further justification. How do these choices affect the model's performance? Additionally, exploring other feature engineering techniques or data augmentation strategies could potentially improve the model's robustness and generalizability.**

**Response:**

Thank you for your meticulous review and valuable suggestions regarding our experimental setup! You have rightly pointed out the importance of detailing hyperparameters and training specifics, particularly how window size and the number of factors in factor analysis impact model performance. Addressing these concerns is essential for enhancing the reproducibility of our experiments and the robustness of our model.

1. **Window Size Selection:** In this study, we set the window size to 64 seconds. We chose this window size based on the characteristics of EEG signals and the typical time range in which pre-ictal signs of epilepsy appear, usually within minutes to tens of minutes. A 64-second window can capture sufficient temporal context information while avoiding overly long windows that might dilute features or overly short windows that might lose context. In preliminary experiments, we compared multiple window sizes (e.g., 32 seconds, 64 seconds, and 128 seconds). The results showed that a 64-second window achieved a good balance between model accuracy, sensitivity, specificity, and computational complexity.
2. **Number of Factors in Factor Analysis:** EEG signals have high dimensionality after preprocessing, so we used factor analysis for linear dimensionality reduction, retaining the primary information related to epileptic seizures. To optimize feature representation, we explored different numbers of factors in our experiments. We found that 64 factors struck a good balance between preserving the main characteristics of the signal and reducing noise. Fewer factors might lead to insufficient features, while too many factors could introduce redundancy, decreasing the model's generalization ability.
3. **Feature Engineering and Data Augmentation:** To further enhance the robustness and generalization of the model, we combined traditional signal processing methods with data-driven techniques. We applied Short-Time Fourier Transform (STFT) and filtering to the EEG signals. These methods effectively preserve the time-frequency characteristics of the signal while reducing noise interference, thereby improving the model's performance on different datasets.

**We have updated Section 2.3 Data Preprocessing in the paper to reflect these details:**

In this study, we chose a 64-second window size, considering the characteristics of EEG signals and the fact that epileptic seizure precur-sors typically occur within a time range of several minutes to tens of minutes. As depicted in Figure 3, a window size of 64 seconds was em-ployed, indicating that each data segment spanned a 64-second duration. A 64-second window effectively captures sufficient temporal context in-formation while avoiding the drawbacks of overly long windows that di-lute features, or overly short windows that lose context. By comparing different window sizes (such as 32 seconds, 64 seconds, and 128 seconds), we found that the 64-second window strikes a good balance between model accuracy, sensitivity, specificity, and computational complexity.

Given the high dimensionality of EEG signals after preprocessing, we applied factor analysis for linear dimensionality reduction to retain the primary information related to epileptic seizures. Through experimenta-tion to optimize the number of factors, we found that 64 factors achieved a good balance between preserving the main features of the signal and reducing noise, avoiding the issue of insufficient features when the factor number is too low or introducing redundancy when it is too high, which could affect the model's generalization ability.

Additionally, to improve the model's robustness and generalization capability, we combined traditional signal processing methods with data-driven techniques. We applied Short-Time Fourier Transform (STFT) and filtering to the EEG signals, preserving the time-frequency characteristics of the signals while reducing noise interference. This approach enhanced the model's performance across different datasets.

**4. The manuscript mentions using a random seed, 5-fold cross-validation, batch size, and the Adam optimizer. However, it lacks a comprehensive discussion of the hyperparameter tuning process. What other hyperparameters were tuned, and what strategies were used for optimization? Providing more details about the training process and hyperparameter selection would enhance the reproducibility of the study.**

**I suggest providing a table or expanded section on training configurations and detailed rationale for the selected hyperparameters would aid clarity and reproducibility.**

**Response:**

Thank you for your valuable suggestion! You have rightly pointed out that more detailed information on the training process and hyperparameter selection is needed. To ensure that the model learns various features in a balanced manner and avoids overfitting or underfitting, we made careful adjustments and optimizations in several aspects. Specifically:

To address potential overfitting or underfitting in multimodal models, we implemented a series of regularization techniques and hyperparameter tuning methods during the experiments. In the CNN module, we used Batch Normalization to accelerate training and improve the model's generalization ability. The choice of learning rates also reflected our understanding of the characteristics of each module. For the CNN part, we adopted an initial learning rate of 0.0001, which helps stabilize the convergence of local features and avoids underfitting caused by overly rapid convergence. After pooling and dimensionality reduction through fully connected layers, the CNN module collaborates with the GRU and Transformer for global feature modeling. For the Sparse Transformer module, we specifically optimized the number of attention heads and encoder layers, using 2 encoder layers and 4 attention heads. This is a trade-off choice based on the balance between performance and computational complexity, aiming to prevent overfitting of global features.

**Thank you for your correction; the following content will be added to the first paragraph of Section 3.1 Evaluation of Prediction Performance:**

To mitigate potential overfitting or underfitting in multimodal mod-els, a range of regularization techniques and hyperparameter tuning methods were applied in the experiments. In the CNN module, Batch Normalization was employed to accelerate training and improve the model's generalization ability. The choice of learning rate also reflects an understanding of each module's characteristics. For the CNN component, an initial learning rate of 0.0001 was selected, which helps stabilize the convergence of local features and prevents underfitting caused by exces-sively fast convergence. After pooling and dimensionality reduction through fully connected layers, the CNN module works in conjunction with global features from the GRU and Transformer modules for joint modeling. In the Sparse Transformer module, special attention was paid to optimizing the number of attention heads and encoder layers. A 2-layer encoder with 4 attention heads was chosen, striking a balance be-tween performance and computational complexity, with the goal of pre-venting overfitting of global features.

**5. Although the results indicate high performance, the statistical significance of these results should be strengthened. The model achieves high accuracy, the evaluation could be more comprehensive. Consider including other relevant metrics like precision, recall, and F1-score to provide a more complete picture of the model's performance. The comparison with other methods is limited. Including a broader range of recent deep learning approaches for seizure prediction and benchmarking against them would strengthen the evaluation. Reporting confidence intervals or statistical tests on the results (especially for key metrics like sensitivity and specificity) would make the results more robust.Although the results show excellent performance, statistical significance needs to be further strengthened. It is recommended to include other relevant metrics such as precision, recall, and F1 score to provide a more comprehensive description of the model's performance.**

**Response:**

Thank you for your valuable suggestion! Your feedback on enhancing the statistical significance of our model evaluation is timely and constructive, especially concerning the need for a more comprehensive performance assessment. We fully agree with your opinion and have improved the experimental results accordingly to provide readers with a more thorough and accurate performance evaluation.

To further strengthen the statistical significance of the model's performance evaluation, we conducted more comprehensive statistical analyses on the CHB-MIT and SWEC-ETHZ datasets. By performing independent sample t-tests on data from 6 patients in each dataset, the results showed no statistically significant differences between the two groups in ACC (CHB-MIT: 99.23±0.75 vs. SWEC-ETHZ: 99.07±0.52), AUROC (0.970±0.051 vs. 0.981±0.017), sensitivity (98.92±0.89 vs. 98.38±0.99), and F1 score (98.95±0.86 vs. 99.09±0.57) (p > 0.05). This not only validates the stability of the model's performance but also indicates its good generalization ability across different datasets.

Additionally, we supplemented recall as a complementary metric to sensitivity and used the F1 score to comprehensively evaluate the model's precision and recall. The results show that the model achieved over 98% excellent performance on both datasets. These multidimensional statistical analyses further demonstrate the reliability and stability of the CGTNet model in epilepsy prediction tasks. By establishing such a comprehensive performance evaluation system, we not only showcase the model's excellent performance but also ensure the statistical significance of the research results.

**Thank you for your correction; we have made corresponding modifications in the text, and the following content will be added to the last paragraph of Section 3.1:**

To further strengthen the statistical significance of our model evalua-tion, we conducted a more thorough statistical analysis on both the CHB-MIT and SWEC-ETHZ datasets. By performing independent samples t-tests on data from six patients in each dataset, the results revealed no sta-tistically significant differences between the two datasets in terms of ac-curacy (ACC: CHB-MIT 99.23±0.75, SWEC-ETHZ 99.07±0.52), AUROC (0.970±0.051 vs. 0.981±0.017), sensitivity (98.92±0.89 vs. 98.38±0.99), and F1 score (98.95±0.86 vs. 99.09±0.57) (p > 0.05). This finding confirms the sta-bility and generalization capability of the CGTNet model.Additionally, we included recall as a supplementary metric to sensitivity and evaluat-ed the model’s precision and recall through the F1 score. For both da-tasets, the model's F1 score exceeded 98%, further demonstrating its ex-cellent classification performance.

Through these multidimensional statistical analyses, we not only highlight the model's outstanding performance but also ensure the statis-tical significance of our findings, thus further validating the reliability and robustness of CGTNet in epilepsy seizure prediction.

**6. While the model is tested on two datasets, a discussion of generalization to other EEG data sources or patient demographics is missing. The manuscript briefly mentions the clinical relevance of the study. However, it needs a more detailed discussion of how the model can be translated into clinical practice. What are the potential challenges and limitations in real-world applications? How can the model be integrated into existing clinical workflows for epilepsy diagnosis and management? Addressing these questions would enhance the impact and significance of the study. Adding a discussion on the potential limitations of CGTNet in clinical applications, along with suggestions for future work to enhance generalizability, would be valuable.**

**Response:**

Thank you for your valuable comments! You have highlighted important aspects regarding the generalizability of EEG data sources and patient demographics, as well as the challenges in translating the model into clinical practice. We greatly appreciate your suggestions and have supplemented and discussed these points in the paper.

Regarding the generalizability of EEG data and clinical translation, we have conducted in-depth thinking. The CHB-MIT dataset used in this study includes 22 patients (5 males, ages 3–22; 17 females, ages 1.5–19), and we also included data from 6 patients from the SWEC-ETHZ dataset, covering patients of different ages and genders. This diverse data source helps the model establish more universal feature representations. However, we recognize that translating the CGTNet model into clinical practice still faces several key challenges:

Firstly, EEG data in clinical environments may be subject to more noise interference, such as patient movement and equipment artifacts, requiring the model to have stronger anti-interference capabilities. Secondly, different medical institutions may use varying EEG equipment, necessitating the model to adapt across devices. Real-time prediction systems need to operate efficiently under limited computational resources, posing requirements for model lightweighting.

To address these challenges, our future work will focus on: (1) Expanding the scale and diversity of validation datasets to validate the model on a larger and more varied patient population; (2) Investigating model adaptation mechanisms to enhance the model's ability to adapt to data characteristics in different clinical environments; (3) Optimizing the model structure to improve computational efficiency while maintaining predictive performance, making it more suitable for operation in resource-constrained clinical environments.

Additionally, we will explore integration schemes of the model into medical devices and how to better apply it in practical clinical scenarios while protecting patient privacy.

**Thank you for your correction; the following content will be added to the conclusion of the paper:**

This study utilized the CHB-MIT and SWEC-ETHZ datasets, which include data from 22 patients (5 males, aged 3-22 years; 17 females, aged 1.5-19 years) and 6 additional patients from the SWEC-ETHZ dataset. These datasets, which encompass patients of various ages and genders, enable the model to learn more generalizable features. However, apply-ing the model in clinical practice still presents several challenges.

EEG data collected in clinical settings can be affected by noise, such as patient movement or equipment malfunctions, which requires the model to be more resilient to such disturbances. Additionally, EEG de-vices used across different hospitals can vary, making it essential for the model to be adaptable to different devices. Real-time prediction systems must also operate efficiently in resource-constrained environments, which means the model must be lightweight.

Future work will focus on: (1) expanding the dataset to validate the model across a more diverse patient population; (2) improving the mod-el's adaptability to handle data from various clinical settings; and (3) op-timizing the model's architecture to strike a balance between prediction accuracy and computational efficiency, ensuring it is suitable for re-source-limited environments. Furthermore, we will explore potential in-tegration solutions for embedding the model in medical devices to ensure its practical application in clinical scenarios, while safeguarding patient privacy.

**Minor Issues:**

1. **The manuscript could benefit from careful proofreading to correct minor grammatical errors and improve clarity.**

We have proofread and corrected grammatical errors.

2. **Ensure consistency in referencing style throughout the manuscript.**

We have confirmed and made necessary adjustments.

3. **Some figures (e.g., Figure 6) could be improved in terms of resolution and clarity.**

We will upload high-definition vector images. Please let us know if further adjustments are needed.

4. **More recent advancements in epilepsy prediction using deep learning and attention mechanisms seem to be missing. Updating the literature review would improve the paper’s background section.**

This content has been supplemented.

Above are our responses and corresponding modifications to each of your comments. Once again, we sincerely thank you for your valuable suggestions and guidance during the review process. Your input has greatly helped us improve the paper. If you have any further suggestions or questions, please feel free to let us know. We look forward to your re-evaluation and hope the final version meets your expectations.
