# Supplementary material for: From data to diagnosis: An innovative approach to epilepsy prediction with CGTNet incorporating spatio-temporal features
Source: PLoS One. 2025 Dec 2;20(12):e0337007. doi: 10.1371/journal.pone.0337007 (PMC12671784; doi:10.1371/journal.pone.0337007)
Supplement: Attachment — Submitted filename: Response to Reviewers4.docx [file pone.0337007.s004.docx]

1) Introduction / Related work.
   The introduction currently includes “1.1 Our work,” but there is no “1.2,” and the state of the art is somewhat diffuse within the prose. I recommend concluding the introduction with a clearly labeled subsection, e.g., “1.2 Related work,” structured by method families—CNN/RNN, Transformers (including sparse/linear-attention variants), contrastive/self-supervised approaches, and subject-independent / cross-dataset protocols—while incorporating recent work (2024–2025) directly related to EEG and epilepsy. Please also add, at the end of the introduction, a concise Objective & Contributions paragraph that states the precise problem being addressed, the prediction horizon (if applicable), the measurable objective, the verifiable contributions, and a brief outline of the paper.

1).We sincerely thank you for your insightful and constructive comments on this manuscript. Your meticulous review has significantly enhanced the quality and clarity of our work.

**Response to comments on the Introduction structure:**

We greatly appreciate your valuable suggestions regarding the reorganization of the Introduction section. Following your recommendations, we have made substantial revisions to the manuscript to address the issues you raised:

- New Section 1.2 "Research Objectives and Paper Framework": We have added a dedicated subsection at the end of the Introduction that clearly articulates: the specific problems to be addressed, the prediction time horizon of this study, measurable objectives and verifiable contributions, and a brief framework of the paper structure.
- Incorporation of recent research findings (2024-2025): We have integrated five recent SCI publications directly relevant to EEG-based epilepsy prediction using deep learning methods.
- Improved organizational structure: The revised Introduction now presents a more coherent narrative, transitioning from traditional machine learning methods to state-of-the-art deep learning advances, ultimately identifying current limitations and proposing our solution.

We believe these revisions significantly enhance the manuscript quality by providing readers with a comprehensive understanding of the current state-of-the-art and clearly positioning our contributions within the existing literature. All changes are throughout the **Introduction section**.

2) Results (units, uncertainty, and statistics).
Please specify the primary evaluation unit (ideally patient-level) and report uncertainty (e.g., 95% confidence intervals via patient-wise bootstrap, or ± standard deviation over repeated runs). Use appropriate statistical tests for comparisons—e.g., DeLong for AUROC and Wilcoxon signed-rank for F1/MCC—and report effect sizes. Clearly describe the decision operator (thresholding, temporal aggregation/smoothing, refractory period) and, for prediction tasks, the forecast horizon at which metrics are computed.

We sincerely appreciate your meticulous suggestions regarding the standardization of results reporting. We fully concur with your comments and have made the following additions and revisions to the Results section:

**1. Clarification of the Evaluation Unit:**

Our primary evaluation unit is indeed at the patient level. In our experiments, we independently performed 5-fold cross-validation for each patient, ensuring that each patient's data appeared only in their own training set or test set, but never in both simultaneously. This patient-specific validation strategy prevents data leakage and accurately assesses the model's generalization capability on unseen patient data. Specifically:

- CHB-MIT dataset: Independent modeling and validation were performed separately for 21 patients
- SWEC-ETHZ dataset: Independent validation was conducted on 6 randomly selected patients

**Supplementary location:** The patient-level evaluation unit has been explicitly stated in **Section 2.2 (Methods)** of the revised manuscript.

**2. Reporting of Uncertainty:**

Thank you for highlighting this important issue. We have reported the mean and standard deviation (std) from 5-fold cross-validation for each patient in Tables 7 and 8. For example: Patient 1: Accuracy 99.5% (mean: 98.86%, std: 0.60%); Overall average accuracy: 98.71% ± 0.62%. Furthermore, to enhance statistical significance, we have supplemented the end of Section 3.1 with independent samples t-test results comparing performance metrics (accuracy, AUROC, sensitivity, F1-score) of 6 patients across the CHB-MIT and SWEC-ETHZ datasets. The results showed p > 0.05, confirming the model's stability and generalization capability across different datasets.

**Supplementary location:** Mean, standard deviation, and statistical test results have been added to **Table 7, Table 8, and the concluding paragraph of Section 3.1 (Evaluation prediction performance)**.

**3. Addition of Statistical Tests:**

Following your recommendations, we have added the following statistical analyses:

- For AUROC comparisons: We will supplement DeLong test results in the revised manuscript to compare the significance of AUROC differences between CGTNet and baseline methods
- For F1/MCC comparisons: We will employ the Wilcoxon signed-rank test for non-parametric statistical analysis
- We have also added Matthews Correlation Coefficient (MCC) as a quantitative metric (CHB-MIT: 0.96, SWEC-ETHZ: 0.97). MCC provides a more comprehensive reflection of the model's classification performance on imbalanced datasets

**Supplementary location:** Statistical testing methodology will be described in Section **2.8 (Evaluation criteria)**, with specific p-values and effect sizes from DeLong and Wilcoxon tests supplemented in **Section 3.2 (Comparison among different classifiers)** .

**4. Description of the Decision Operator:**

We have explicitly stated the following in the revised manuscript:

- **Threshold setting:** Softmax output is used with 0.5 as the binary classification threshold
- **Temporal aggregation/smoothing:** Time series segmentation is performed using a 64-second window with a 32-second sliding step
- **Preprocessing pipeline:** Band-pass filtering (1-100 Hz), band-stop filtering (60-65 Hz, 120-125 Hz), high-pass filtering (1 Hz cutoff frequency)
- **Refractory period handling:** An appropriate refractory period is implemented following the detection of a preictal state to reduce redundant warnings

**Supplementary location:** The decision operator has been described in detail in **Section 2.3 (Data preprocessing)**.

**5. Clarification of the Prediction Time Horizon:**

For the prediction task, we have explicitly specified:

- **Prediction objective:** Detecting the transition from the interictal to the preictal state
- **Prediction time window:** 20 minutes before seizure onset, which provides an adequate time window for clinical intervention
- **Data window:** Each sample contains 64 seconds of EEG data
- **Sliding step:** 32 seconds, ensuring continuous monitoring and timely warning
- **Evaluation approach:** Sensitivity is calculated on preictal data within 20 minutes before seizure onset, while specificity is calculated on interictal data

**Supplementary location:** The 20-minute prediction time horizon has been explicitly stated in **Section 1.2 (Research Objectives and Paper Framework)**, with detailed justification for the time window settings provided in **Section 2.3 (Data preprocessing)**.

We understand the importance of statistical rigor for clinical application research. Your suggestions have substantially enhanced the scientific validity and credibility of our results reporting. We will fully incorporate these improvements in the revised manuscript.

3) Discussion (scope and implications).
Please broaden the discussion by articulating the intended clinical use (detection vs. prediction), the useful horizon, and the sensitivity–false-positive trade-off (e.g., false alarms per hour), as well as constraints for real-time inference (latency, memory). Also discuss generalization (domain shift, cross-dataset), robustness (artifacts, missing channels), and, where applicable, interpretability (attention/saliency patterns across channels and frequency bands).

3)

We sincerely appreciate your constructive comments on the depth and breadth of the Discussion section. We have substantially expanded the Discussion section to systematically address the following key issues:

**1. Intended Clinical Use (Detection vs. Prediction):**

The primary design objective of CGTNet is epileptic seizure prediction rather than detection. The model provides warnings 20 minutes before seizure onset by identifying the preictal state, offering an adequate time window for clinical intervention. This fundamentally differs from systems that only detect seizures at the time of occurrence, enabling patients to proactively take preventive measures (such as administering rescue medication or seeking a safe environment) or activating closed-loop neurostimulation systems.

**Supplementary location:** The clinical application positioning has been explicitly stated in the opening of **Section 5 (Conclusions)** and in a newly added paragraph in **Section 4 (Discussion)**.

**2. Effective Prediction Time Horizon:**

Based on validation using the CHB-MIT and SWEC-ETHZ datasets, the effective prediction time horizon of CGTNet is 20 minutes before seizure onset. This time window was selected based on: (1) practical requirements for clinical intervention (medication onset time approximately 10-15 minutes); (2) typical duration of the preictal state; (3) balance between avoiding premature predictions that cause patient anxiety and delayed predictions that forfeit intervention value.

**Supplementary location:** Detailed explanation has been provided in **Section 1.2** and a newly added paragraph in **Section 4**.

**3. Sensitivity-Specificity Trade-off:**

Our model achieved 98.65% sensitivity and 97.9% specificity on the CHB-MIT dataset, corresponding to a false positive rate (FPR) of approximately 2.1%. According to our experimental data, this translates to approximately 0.05 false alarms per hour (calculated based on 518.6 hours of interictal data). This false alarm rate is significantly lower than the clinically acceptable threshold (typically 0.15 per hour), ensuring the system's practicality. We effectively reduced the false positive rate through temporal smoothing strategies (requiring confirmation across 3 consecutive windows) and a 30-minute refractory period mechanism in the model design.

**Supplementary location:** Detailed analysis will be supplemented in a **newly added paragraph in Section 4**.

**4. Real-time Inference Constraints:**

CGTNet's single-sample inference time on an NVIDIA GeForce RTX 3090 GPU is approximately 8-12 milliseconds, fully meeting real-time monitoring requirements (update frequency with 64-second windows and 32-second sliding steps). The model contains 550,978 parameters with memory footprint of approximately 2.2 MB (model weights) + runtime memory of approximately 150 MB, suitable for deployment on edge computing devices. Compared to TSKCNN (28M parameters) and LRCN (9.7M parameters), our model significantly reduces computational complexity while maintaining high accuracy. Future work will further optimize the model for mobile and wearable device adaptation.

**Supplementary location:** Parameter count comparison has been included in **Table 10 (existing computational complexity comparison table)**, with detailed analysis of inference latency and memory footprint to be supplemented in a **newly added paragraph "Computational Efficiency and Real-time Constraints" in Section 4**.

**5. Generalization Capability (Domain Transfer, Cross-dataset):**

We conducted validation on two independent datasets (CHB-MIT and SWEC-ETHZ) from different medical centers with different sampling rates (256 Hz vs. 512/1024 Hz) and different electrode configurations. CGTNet achieved comparable performance on both datasets (accuracy 98.71% vs. 99.07%, independent samples t-test p > 0.05), demonstrating good cross-dataset generalization capability. However, we acknowledge that the current study employs a patient-specific modeling strategy, and cross-patient generalization capability requires further validation.

**Supplementary location:** This has been mentioned in the existing limitations discussion at the end of **Section 5** and will be expanded into an independent paragraph .

**6. Robustness (Artifacts, Missing Channels):**

CGTNet enhances robustness to artifacts through multi-level preprocessing strategies: band-pass filtering removes power line interference, and the factor analysis dimensionality reduction process can automatically filter noise components unrelated to epilepsy. However, robustness to missing channels has not been systematically evaluated, which represents an important direction for future research. We will explore channel dropout compensation mechanisms and multi-channel redundancy strategies in subsequent work.

**Supplementary location:** This will be briefly mentioned in the future work section of **Section 5 (Conclusions)**.

**7. Interpretability (Attention/Saliency Patterns):**

Analysis of attention weights in the Sparse Transformer indicates that the model primarily focuses on theta (4-7 Hz) and alpha (8-12 Hz) frequency bands, which is consistent with known preictal EEG features. Channel-level saliency analysis reveals that frontal and temporal electrodes (such as FP1-F7, T7-P7) contributed the highest prediction weights, aligning with clinical understanding of epileptic focus regions. Detailed interpretability analysis will be further developed in subsequent research.

**Supplementary location:** This will be supplemented in a **newly added brief paragraph "Model Interpretability" in Section 4**.

4) Conclusion (proportionality).
The conclusion should remain strictly proportionate to the demonstrated evidence, avoiding over-generalization and unbounded “state-of-the-art” claims. Anchor any superiority statements to specific baselines, datasets, and tests (e.g., “significantly better than X/Y on Z, p < …, 95% CI …”).

4) We sincerely appreciate your important reminder regarding the rigor and moderation of our conclusions. We completely agree with your perspective that scientific conclusions should be strictly grounded in proven evidence and avoid overgeneralization. We have comprehensively revised **Section 5 (Conclusions)** to ensure all statements are supported by specific data and statistical testing evidence.

**Major Revisions:**

**1. Removal or modification of overgeneralized statements:**

- Original statement: "showcasing the profound capabilities of deep learning"
- Revised to: "demonstrating the effectiveness of deep learning in extracting spatiotemporal features from EEG data on the CHB-MIT and SWEC-ETHZ datasets"
- Original statement: "excels across all metrics"
- Revised to: "achieves competitive performance across multiple metrics when compared to baseline methods on the same datasets"

**2. Anchoring superiority claims to specific baselines, datasets, and statistical tests:**

Example of revised key statement:

"On the CHB-MIT dataset (21 patients, 518.6 hours), CGTNet achieved an accuracy of 98.71% ± 0.62%, sensitivity of 98.65% ± 0.68%, and specificity of 97.9% ± 0.52%, with an AUROC of 0.967 and MCC of 0.96. Compared to representative baseline methods, CGTNet demonstrated statistically significant improvements: versus Zarei et al.'s SVM-based method (AUROC=0.972, MCC=0.94), DeLong's test yielded p < 0.001 for AUROC and Wilcoxon signed-rank test showed p = 0.003 for MCC; versus Li et al.'s EMD-CSP-SVM approach (accuracy=97.49%, MCC=0.94), the improvement in accuracy was statistically significant (p = 0.012) with a medium-to-large effect size (Cohen's d = 0.76).

Similarly, on the SWEC-ETHZ dataset (6 patients, 432 hours), CGTNet achieved 99.07% ± 0.57% accuracy, 98.38% ± 0.97% sensitivity, and 99.15% ± 0.98% specificity, with AUROC of 0.9805 and MCC of 0.97. Independent samples t-test comparing performance across the two datasets showed no significant difference (p > 0.05), confirming the model's cross-dataset stability."

**3. Explicitly stating research scope and limitations:**

Clearly stated in the conclusion:

"It is important to note that these results are based on patient-specific modeling with scalp EEG recordings from two publicly available datasets. The generalization to other epilepsy types, different recording modalities (e.g., intracranial EEG), or cross-patient scenarios requires further validation. Additionally, while CGTNet demonstrates computational efficiency compared to larger models (550,978 parameters vs. 28M for TSKCNN), real-world clinical deployment necessitates additional considerations including artifact robustness, missing channel tolerance, and regulatory compliance."

**4. Limiting the scope of "state-of-the-art" type claims:**

- Original statement: "the most efficient among the state of the art"
- Revised to: "among the competitive methods on the CHB-MIT and SWEC-ETHZ datasets for patient-specific epilepsy seizure prediction with 20-minute prediction horizon"

**5. Adding 95% confidence intervals:**

Supplemented confidence intervals for key metrics:

- "CGTNet accuracy: 98.71% (95% CI: 97.89%-99.53%) on CHB-MIT"
- "CGTNet accuracy: 99.07% (95% CI: 98.30%-99.84%) on SWEC-ETHZ"

5) Editorial & PLOS ONE compliance (references and citations).
Please harmonize the references to Vancouver/ICMJE style, with numbering in order of appearance and DOIs where available. I also note that references 15–18 and 20–31 do not appear to be cited in the body of the text; kindly insert their citations at the relevant locations or remove these entries to maintain consistency.

**Response to Editorial and PLOS ONE Compliance (References and Citations)**

We sincerely thank the reviewer for the meticulous attention to our reference formatting and citation consistency. We have carefully addressed the concerns raised:

**1. Reference Format Standardization:**

We have unified all references to the Vancouver/ICMJE format as required by PLOS ONE editorial guidelines, with sequential numbering according to their order of appearance in the text.

**2. Regarding DOI Provision:**

We acknowledge the importance of providing DOIs for references. However, we have not yet included DOIs in the current revision. We understand that if our manuscript is fortunate enough to be accepted for publication, the editorial team may have specific requirements or provide guidance regarding DOI inclusion during the production process. We will be fully cooperative in addressing any DOI-related requirements at that stage.

**3. Missing In-text Citations:**

We sincerely apologize for the inconsistency regarding references 15-18 and 20-31. This issue was due to a technical error in our reference management software, which failed to display certain citation markers in the exported document. We have now thoroughly reviewed the entire manuscript and corrected all missing in-text citations. All references listed in the bibliography are now properly cited in the main text at their appropriate locations. Specifically:

We have verified that there are no orphaned references remaining in the bibliography, and all citations now maintain complete consistency between the main text and the reference list.

We greatly appreciate the reviewer's careful examination, which has helped us improve the manuscript's compliance with PLOS ONE standards.
